# Electrical Characteristics of the Uniaxial-Strained nMOSFET with a Fluorinated HfO_2_/SiON Gate Stack

**DOI:** 10.3390/ma7032370

**Published:** 2014-03-20

**Authors:** Yung-Yu Chen

**Affiliations:** Department of Electronic Engineering, Lunghwa University of Science and Technology, Guishan, Taoyuan 333, Taiwan; E-Mail: yungyu@mail.lhu.edu.tw or yungyu1975@gmail.com; Tel.: +886-2-8209-3211 (ext. 5610); Fax: +886-2-8209-3211 (ext. 5699)

**Keywords:** fluorine, strain, HfO_2_

## Abstract

The channel fluorine implantation (CFI) process was integrated with the Si_3_N_4_ contact etch stop layer (SiN CESL) uniaxial-strained n-channel metal-oxide-semiconductor field-effect transistor (nMOSFET) with the hafnium oxide/silicon oxynitride (HfO_2_/SiON) gate stack. The SiN CESL process clearly improves basic electrical performance, due to induced uniaxial tensile strain within the channel. However, further integrating of the CFI process with the SiN CESL-strained nMOSFET exhibits nearly identical transconductance, subthreshold swing, drain current, gate leakage and breakdown voltage, which indicates that the strain effect is not affected by the fluorine incorporation. Moreover, hydrogen will diffuse toward the interface during the SiN deposition, then passivate dangling bonds to form weak Si-H bonds, which is detrimental for channel hot electron stress (CHES). Before hydrogen diffusion, fluorine can be used to terminate oxygen vacancies and dangling bonds, which can create stronger Hf-F and Si-F bonds to resist consequent stress. Accordingly, the reliability of constant voltage stress (CVS) and CHES for the SiN CESL uniaxial-strained nMOSFET can be further improved by the fluorinated HfO_2_/SiON using the CFI process. Nevertheless, the nMOSFET with either the SiN CESL or CFI process exhibits less charge detrapping, which means that a greater part of stress-induced charges would remain in the gate stack after nitrogen (SiN CESL) or fluorine (CFI) incorporation.

## 1. Introduction

According to the international technology roadmap of semiconductors (ITRS), the equivalent oxide thickness (EOT) of the gate stack for metal-oxide-semiconductor field-effect transistors (MOSFETs) has to be scaled gradually to fulfill and increase device performance [1]. However, a drastically increasing direct tunneling leakage current in thin gate dielectrics cannot be tolerated for low standby power or low operation power applications. High-permittivity (high-k) metal oxides are thought to be gate dielectric materials for silicon (Si)-based devices, since a larger physical thickness than silicon dioxide (SiO_2_) or silicon oxynitride (SiON) can be utilized to reduce gate leakage current by suppression of direct tunneling, while maintaining required specific gate capacitance [2–6]. For these reasons, various high-k dielectrics, including yttrium oxide (Y_2_O_3_), zirconium oxide (ZrO_2_), lanthanum oxide (La_2_O_3_) and hafnium oxide (HfO_2_) have been extensively studied as alternative gate dielectrics [2–8].

Among these high-k dielectrics, HfO_2_ and ZrO_2_ are the greatest potential candidates under investigation, due to the high dielectric constant, wide band gap and high conduction band offset with respect to Si [9]. Although ZrO_2_ has similar properties to HfO_2_, interface reaction between ZrO_2_ and Si has been acknowledged [10]. Consequently, HfO_2_ has been chosen as the gate dielectric. Unfortunately, bias temperature instability (BTI) becomes a serious reliability problem for deep-submicron MOSFETs with high-k gate dielectrics, due to high bulk and interfacial defect densities [11,12]. Moreover, high defect densities within the gate stack would also increase scattering probability for channel carriers, and result in mobility degradation and drain current reduction [2,13]. Hence, strain technology has been successfully integrated with current complementary metal-oxide-semiconductor (CMOS) technology in order to increase both carrier mobility and drain current.

Biaxial or uniaxial strain has been demonstrated to improve carrier mobility in recent years [14]. A strain-Si/SiGe channel was originally proposed to drastically improve both electron and hole mobility [15,16]. However, the integration of the biaxial strain process with existing CMOS technology has become complicated and not easily reduced in cost. Various uniaxial strain technologies have been therefore developed to improve carrier mobility, such as strain source/drain, a high stress Si_3_N_4_ contact etch stop layer (SiN CESL) and stress memorization technology [17–20]. By controlling the stress type of the CESL film on the channel region, for instance, using tensile stress for n-channel MOSFET (nMOSFET), while using compressive stress for p-channel MOSFET (pMOSFET), SiN CESL becomes the simplest process to improve carrier mobility among these uniaxial strain technologies. However, a large amount of hydrogen during SiN CESL deposition would diffuse into the gate stacks to from Si-H/Hf-H bonds. The binding energy of the Si-H/Hf-H bonds is too small to resist subsequent channel hot electron stress (CHES) or constant voltage stress (CVS), which results in a considerable threshold voltage (*V*_TH_) shift and reliability degradation for the SiN CESL uniaxial-strained MOSFETs [21,22].

**Figure 1.**
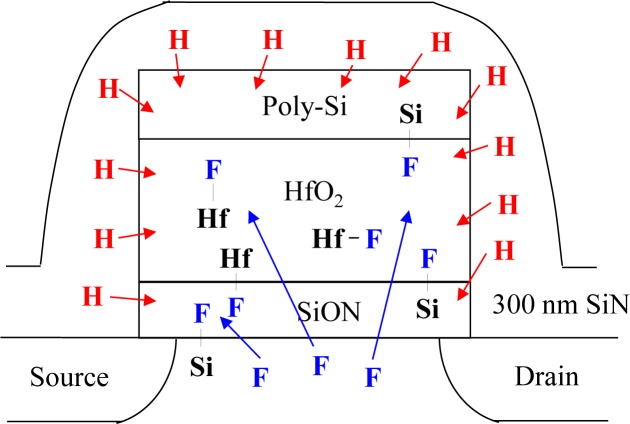
A schematic cross-section of the Si_3_N_4_ contact etch stop layer (SiN CESL) uniaxial-strained n-channel metal-oxide-semiconductor field-effect transistor (nMOSFET) with a fluorinated HfO_2_/SiON gate stack using the channel fluorine implantation (CFI) process.

Furthermore, the dielectric properties and device reliabilities of the MOSFETs can be improved by fluorine incorporation processes, such as fluorine plasma treatment, channel fluorine implantation (CFI), source/drain region fluorine implantation and fluorinated silicate glass (FSG) passivation [23–27]. Incorporating fluorine within high-k gate stacks can terminate interfacial dangling bonds and bulk oxygen vacancies during subsequent processes, which is useful to reduce gate leakage current and improve the charge-to-breakdown and *V*_TH_ instability, as well [28]. Although fluorine passivation technology has been widely used to replace weak Si-H bonds within the high-k gate stack to improve stress reliability, the impact of combining the fluorine incorporation effect with the SiN CESL-strained nMOSFET has not been fully investigated. Therefore, in this paper, fluorine incorporation using the CFI process has been used to comprehensively evaluate the electrical performance and device reliability of the SiN CESL uniaxial-strained nMOSFET with the fluorinated HfO_2_/SiON gate stack, which is expected to reduce *V*_TH_ shift during both CVS and CHES, while maintaining a high drain current. Figure 1 presented the schematic cross-section of the SiN CESL strained nMOSFETs with a fluorinated HfO_2_/SiON gate stack using the CFI process, where fluorine passivation is indicated. A control device without applying both CFI and SiN CESL processes was also prepared for comparison.

## 2. Results and Discussion

**Figure 2.**
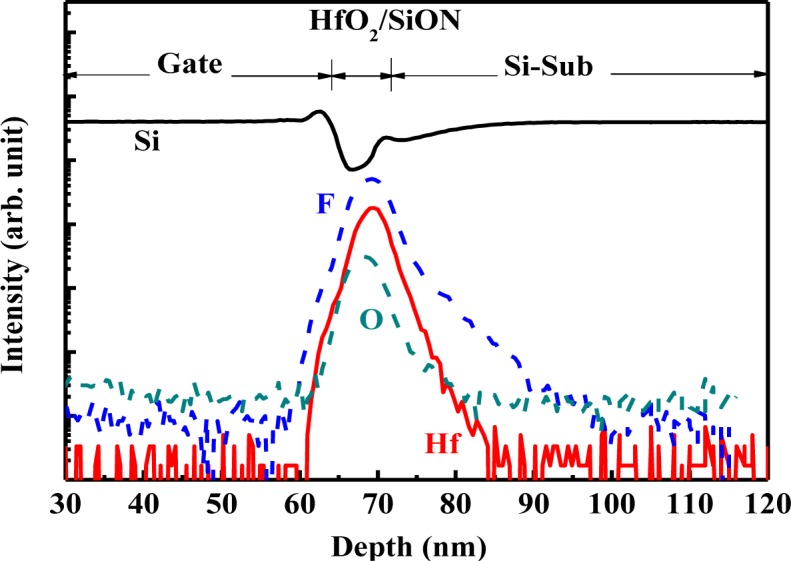
The secondary-ion mass spectroscopy (SIMS) depth profile of the SiN CESL-strained nMOSFET with a fluorinated HfO_2_/SiON gate stack.

**Figure 3.**
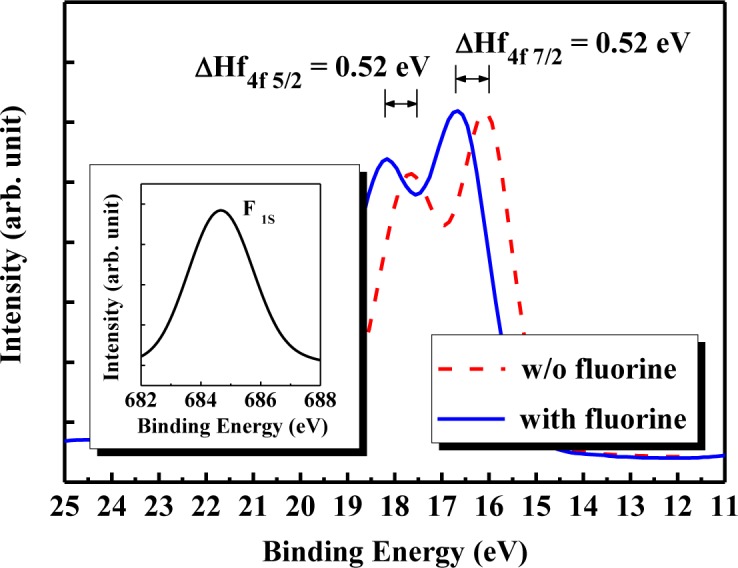
The XPS analysis of the Hf_4f_ electronic spectra for the SiN CESL-strained nMOSFET with and without fluorine incorporation. The F_1s_ signal of the fluorinated HfO_2_/SiON gate stack using the CFI process is also shown in the inset.

Figure 2 shows the SIMS depth profile of the HfO_2_/SiON gate-stacked nMOSFET with CFI and SiN CESL. Although the fluorine atoms have been implanted into a silicon substrate before the dielectric deposition, the result obviously indicates that fluorine can be out-diffused from the substrate and effectively incorporated into the HfO_2_/SiON gate stack during the subsequent high temperature process, which is helpful for passivating the oxygen vacancies to form stronger Hf-F bonds, and therefore, reducing charge trapping [28]. Moreover, the peak of fluorine concentration gathers closer to the bottom interface. As a result, fluorine can pile up at the interface between the HfO_2_/SiON gate stack and the Si substrate, which also reveals that the CFI process exhibits a high probability of terminating interfacial dangling bonds to create robust Si-F bonds to resist subsequent reliability stress. The XPS spectra of the Hf_4f_ signal for the HfO_2_/SiON gate stack with and without the CFI process is displayed in Figure 3. Detected binding energy is calibrated by the C_1s_ signal at 284.5 eV. Compared with un-fluorinated dielectrics, the fluorinated gate stack clearly increases the binding energy more than 0.5 eV for both the Hf_4f5/2_ and Hf_4f7/2_ signals. The binding energy of the Hf_4f5/2_ signal increases from 17.64 eV to 18.16 eV, while the binding energy of the Hf_4f7/2_ signal increases from 16.14 eV to 16.66 eV. Fluorine incorporation into the HfO_2_/SiON gate stack is further confirmed due to a conspicuous signal at ~685 eV, as shown in the inset, which means that the fluorine has been successfully bonded to the HfO_2_.

**Figure 4.**
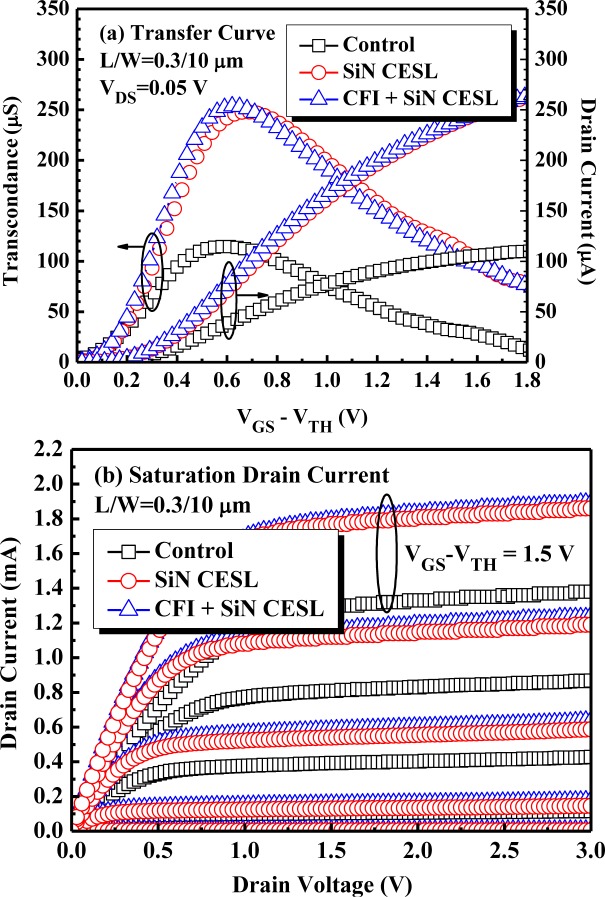
(**a**) Transfer curve and (**b**) saturation drain current of the nMOSFETs with an HfO_2_/SiON gate stack.

Figure 4a presents the transfer curve and extracted transconductance of nMOSFETs measured with a small drain voltage (*V*_DS_ = 0.05 V). The maximum transconductance (*G*_m_) of the control and SiN CESL-strained nMOSFET are 115 μS and 248 μS, respectively. Compared to the control device, the SiN CESL-strained nMOSFET obviously improves *G*_m_ more than 116%, due to a high uniaxial tensile strain [21]. Moreover, a large amount of hydrogen-based species during the SiN CESL deposition would diffuse into the interface between the gate stack and the Si substrate and then passivate interfacial dangling bonds. Therefore, the SiN CESL-strained device can significantly improve the subthreshold swing (*SS*) from 108 mV/dec to 97 mV/dec. Further combining the CFI process with the SiN CESL-strained nMOSFET only slightly improves *G*_m_ and *SS* to 254 μS and 96 mV/dec, respectively. The saturation drain current (*I*_DSsat_) of the nMOSFETs is compared in Figure 4b. Compared with the control device, the SiN CESL-strained device clearly increases *I*_DSsat_ more than 36%, while combining the CFI process with the SiN CESL-strained nMOSFET further increases the *I*_DSsat_ by more than 38%.

**Figure 5.**
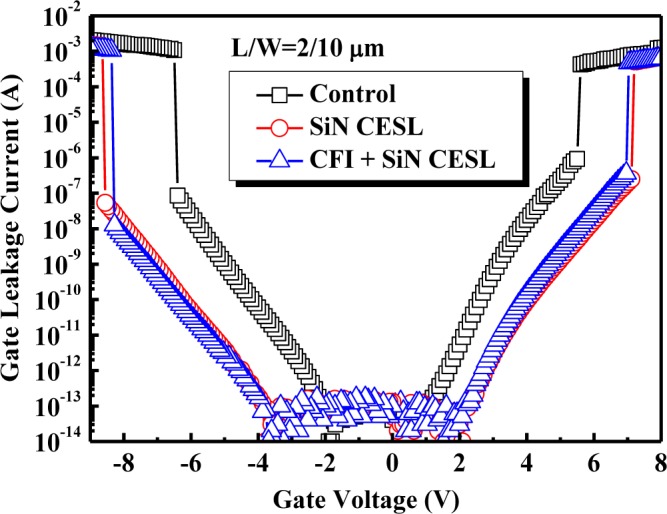
The gate leakage current characteristic of the nMOSFETs with an HfO_2_/SiON gate stack.

Figure 5 plots the gate leakage current characteristics of the nMOSFETs under the positive (inversion) and negative (accumulation) polarity. Compared with the control device, reduced gate leakage current, as well as an increased breakdown voltage has been observed for the SiN CESL-strained device with or without gate stack fluorination. Since gate leakage current and breakdown voltage strongly depend on defect densities, a superior gate insulating property of the nMOSFETs with the SiN CESL and CFI process can be primarily attributed to defect density reduction (e.g., dangling bonds and oxygen vacancies) by hydrogen/nitrogen and fluorine atoms, respectively [19,27]. Although the fluorinated high-k dielectrics widely exhibits superior dielectric characteristics, due to the reduction of the oxygen vacancies and dangling bonds [27,28], further combining of the CFI process negligibly improves the as-fabricated electrical performance of the SiN CESL-strained nMOSFET in this paper, including transconductance, drain current, subthreshold swing, gate leakage current and breakdown voltage. Consequently, stress-induced drain current enhancement has been proven not to be affected by the fluorine incorporation.

**Figure 6.**
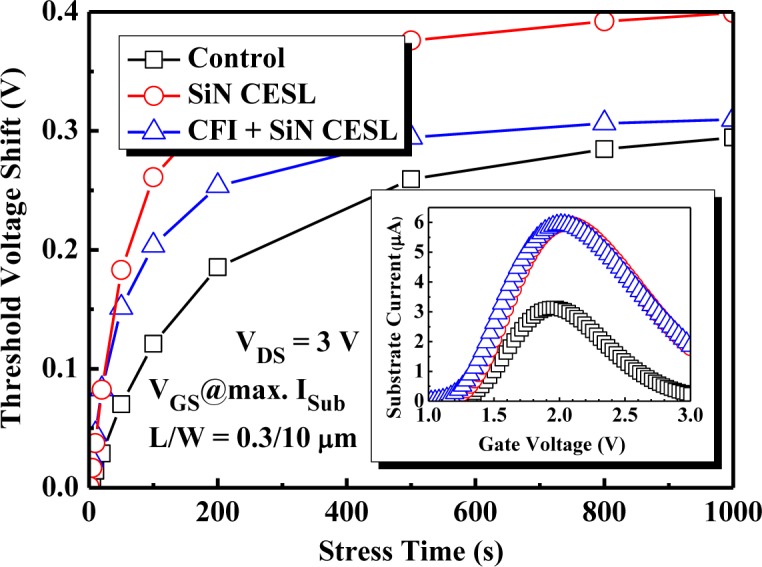
The channel hot electron stress-induced threshold voltage shift of the nMOSFETs with an HfO_2_/SiON gate stack under the maximum substrate current (*I*_sub_) bias condition. The substrate current is also shown in the inset.

Figure 6 exhibits the *V*_TH_ shift of the nMOSFETs during CHES at a maximum substrate current (*I*_sub_). The substrate current during the CHES is also shown in the inset. Obviously, the substrate current of the SiN CESL-strained nMOSFETs with and without the CFI process is much larger than the control device. Although the high tensile stress SiN layer could be used to increase the channel mobility, the substrate current also drastically increases, due to an apparently increased drain current [22], which is demonstrated in Figure 4b. During the SiN layer deposition, a large amount of hydrogen will diffuse into the interface between the gate stack and Si substrate and form weak Si-H bonds, which are easily broken under the subsequent stress and result in a significant *V*_TH_ shift. Moreover, a stressed gate voltage (*V*_GS_) at the maximum *I*_sub_ for the SiN CESL-strained nMOSFETs is larger than the control device, which also partially contributes to a larger CHES-induced *V*_TH_ shift. Therefore, the SiN CESL-strained nMOSFET obviously demonstrates a worse *V*_TH_ shift than the control device.

Further incorporating fluorine into the SiN CESL-strained nMOSFET results in a nearly identical substrate current and stressed gate voltage (*V*_GS_), which can infer that the generated hot electron concentration and injection efficiency is also identical for the SiN CESL-strained nMOSFET with and without the CFI process. However, the CHES degradation is not identical. Combining the CFI process with the SiN CESL-strained nMOSFET obviously reduces the *V*_TH_ shift more than 23% after 1000 s of CHES. The CFI process prior to the gate stack fabrication can be used to easily create robust Si-F bonds near the interface, which have a much higher binding energy than Si-H bonds (the binding energy of the Si-F bond (5.74 eV) is much higher than the Si-H bond (<3.11 eV)) [29]. The nMOSFET with the fluorinated gate stack exhibits a much faster *V*_TH_ shift saturation, which, in turn, demonstrates a more robust fluorine passivation effect. Moreover, the fluorine-incorporated nMOSFET possesses a higher critical energy to create interface traps during CHES [30]. Accordingly, the SiN CESL-strained MOSFET with a fluorinated HfO_2_/SiON gate stack using the CFI process is considered beneficial for suppressing the CHES-induced *V*_TH_ shift.

**Figure 7.**
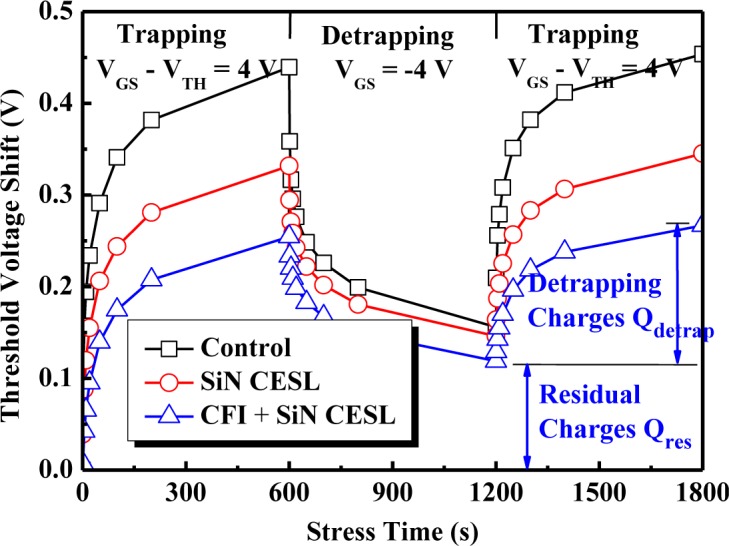
The charge trapping and detrapping behavior of the nMOSFETs with an HfO_2_/SiON gate stack under constant voltage stress.

Figure 7 indicates the charge trapping and detrapping behavior of the nMOSFETs during the CVS, which are stressed (trapped) at 4 V and relaxed (detrapped) at −4 V. The device implemented with either nitrogen (SiN CESL) or fluorine (CFI) incorporation obviously suppresses the *V*_TH_ shift. Since the CVS-induced *V*_TH_ shift is mostly related to bulk traps rather than interface traps, a large amount of nitrogen species would diffuse toward the gate stack and then passivate bulk oxygen vacancies and interfacial dangling bonds, which results in less electron trapping charges (*Q*_trap_) and a small *V*_TH_ shift during the CVS [19]. Moreover, combining the CFI process into the SiN CESL-strained nMOSFET obviously enhances the passivation of the vacancies, due to robust Hf-F bonds formation (6.75 eV), which can further suppress electron trapping and the *V*_TH_ shift during the CVS [29,31]. As a result, combining the CFI process with the SiN CESL-strained nMOSFET is demonstrated to reduce the *V*_TH_ shift during both CVS and CHES, while maintaining a high drain current simultaneously.

**Figure 8.**
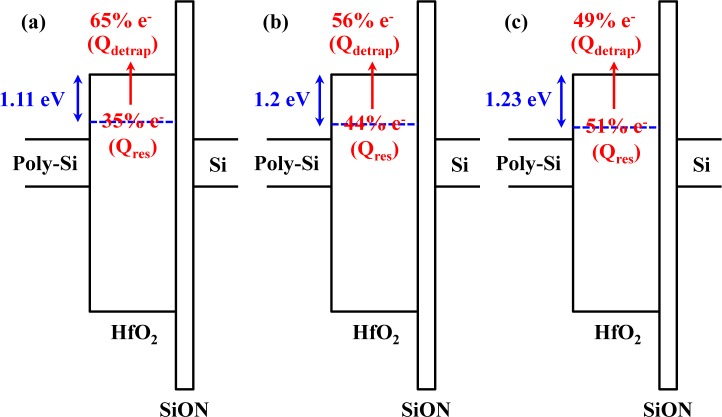
Schematic diagrams of the charge detrapping behavior for (**a**) the control nMOSFET; (**b**) the SiN CESL-strained nMOSFET and (**c**) the SiN CESL-strained nMOSFET with a fluorinated gate stack using the CFI process.

The detrapping charges (*Q*_detrap_), which are extracted from the *V*_TH_ shift during the detrapping period, for the SiN CESL-strained nMOSFETs are less than the control device, implying that a larger part of the CVS-induced charges would remain in the gate stack after nitrogen or fluorine incorporation. After the charge detrapping, 35% of the trapped charges (residual charges, *Q*_res_) remain in the gate stack of the control device. However, 44% and 51% of the trapped charges will remain in the gate stack of the SiN CESL-strained nMOSFET without and with the CFI process, respectively. Fitting with Frenkel–Poole conduction [32], the effective trapping barrier of the control device is 1.11 eV, while the effective trapping barrier of the SiN CESL-strained nMOSFET without and with the CFI process is 1.2 eV and 1.23 eV, respectively. Schematic diagrams of charge detrapping for the gate stack with a shallow trapping barrier (control device) and a deep trapping barrier (SiN CESL-strained nMOSFET with and without the CFI process) are also plotted in Figure 8. Although either nitrogen or fluorine incorporation could effectively improve the CVS reliability, unfortunately, trapped charges within the nitrided or fluorinated HfO_2_/SiON gate stack become more difficult to be detrapped, due to a deeper trapping barrier.

## 3. Experimental

The nMOSFETs were fabricated on 6 in p-type (100) Si wafers with 1–10 Ω-cm resistivity utilizing a conventional self-alignment process. Before 15 nm of sacrificial oxide stripping, fluorinated nMOSFETs were split into CFI at low energy (10 keV) with a 1 × 10^12^ cm^−2^ dosage, followed by the cleaning with a hydrofluoric (HF) acid-last process. A relatively low energy and light dosage was used to mainly prevent significant channel damage and to avoid eliminating SiN CESL-induced tensile strain in the channel. Prior to the high-k gate dielectric deposition, a 0.5-nm interfacial SiON was grown by rapid thermal processing in a nitrous oxide (N_2_O) ambient at 800 °C in order to obtain a SiO_2_-like interface between the high-k dielectric and the Si substrate. A 3-nm HfO_2_ gate dielectric was subsequently deposited by the metal organic chemical vapor deposition system at 500 °C, followed by post deposition annealing at 600 °C in a nitrogen (N_2_) ambient for 30 s to improve the HfO_2_ film quality. A 200-nm poly-Si gate was then deposited by a low-pressure chemical vapor deposition system using silane (SiH_4_) gas at 620 °C.

After typical gate electrode patterning using a lithography stepper and subsequent phosphorous implantation at 25 keV, 5 × 10^15^ cm^−2^, the dopants were activated at 950 °C for 30 s in N_2_ ambient. Afterward, a 300 nm SiN CESL with 370 MPa of tensile strain was deposited using a plasma-enhanced chemical vapor deposition system at 300 °C with SiH_4_ and ammonia (NH_3_). Finally, contact hole etching and aluminum metallization were performed using a standard CMOS process, followed by 400-°C sintering for 30 min.

The electrical properties and reliability characteristics of nMOSFETs with the HfO_2_/SiON gate stack were measured using a semiconductor parameter analyzer. The EOT of the gate stack was extracted from high-frequency (1 MHz) capacitance-voltage (*C*-*V*) curves at strong inversion without considering the quantum effect using a inductance-capacitance-resistance meter. A nearly identical EOT was obtained from all devices, which indicated that both SiN CESL and CFI processes would not cause further interfacial oxidation. Furthermore, the content and distribution of fluorine atoms were measured by secondary-ion mass spectroscopy (SIMS). The binding energy of the hafnium and fluorine atom was extracted from the X-ray photoelectron spectrometer (XPS).

## 4. Conclusions

Fluorine incorporation using the CFI process has been used to comprehensively evaluate the electrical performance and device reliability of the SiN CESL uniaxial-strained nMOSFET with the HfO_2_/SiON gate stack in order to recover the CHES degradation of the CESL-strained device, while maintaining a superior drain current. Basic electrical performance can be drastically improved while introducing the SiN CESL process, due to the induced uniaxial tensile strain within the channel. During the SiN CESL deposition, however, a large amount of hydrogen-based species will diffuse toward the interface and passivate interfacial dangling bonds to form weak Si-H bonds, which is detrimental for CHES. In addition, bulk oxygen vacancies can be terminated by nitrogen atoms during the SiN CESL deposition, which is beneficial for the CVS. An opposite tendency for CVS with respect to CHES for the SiN CESL-strained nMOSFET can be attributed to CVS being mostly related with bulk defects, while CHES is primarily dominated by interfacial defects.

Moreover, the SiN CESL-strained device with a fluorinated HfO_2_/SiON gate stack integrating the CFI process exhibits a nearly identical basic electrical performance. Bulk oxygen vacancies and interfacial dangling bonds can also be bound to fluorine atoms for the nMOSFET with the CFI process and subsequently create stronger Hf-F and Si-F bonds to resist consequent CVS and CHES. Accordingly, both the CVS and CHES reliability of the SiN CESL-strained nMOSFET is further improved by the CFI process. Unfortunately, the nMOSFET, with either the SiN CESL or CFI process, exhibits a smaller charge detrapping ratio, implying that a larger part of the stress-induced charges would remain in the gate stack after nitrogen or fluorine incorporation. The results clearly indicate both detrapping characteristics, and residual charges should be taken into consideration, while implementing the uniaxial strain or fluorine incorporation process on current or future CMOS devices with high-k gate stacks.
